# Adaptive-expert-weight-based load balance scheme for dynamic routing of MoE

**DOI:** 10.3389/fnbot.2025.1590994

**Published:** 2025-10-14

**Authors:** Jialin Wen, Xiaojun Li, Junping Yao, Xinyan Kong, Peng Cheng

**Affiliations:** School of Computer Science, Rocket Force University of Engineering, Xi'an, Shaanxi, China

**Keywords:** mixture of experts, computational optimization, load balancing, routing algorithm, natural language understanding

## Abstract

Load imbalance is a major performance bottleneck in training mixture-of-experts (MoE) models, as unbalanced expert loads can lead to routing collapse. Most existing approaches address this issue by introducing auxiliary loss functions to balance the load; however, the hyperparameters within these loss functions often need to be tuned for different tasks. Furthermore, increasing the number of activated experts tends to exacerbate load imbalance, while fixing the activation count can reduce the model’s confidence in handling difficult tasks. To address these challenges, this paper proposes a dynamically balanced routing strategy that employs a threshold-based dynamic routing algorithm. After each routing step, the method adjusts expert weights to influence the load distribution in the subsequent routing. Unlike loss-function-based balancing methods, our approach operates directly at the routing level, avoiding gradient perturbations that could degrade model quality, while dynamically routing to make more efficient use of computational resources. Experiments on Natural Language Understanding (NLU) benchmarks demonstrate that the proposed method achieves accuracy comparable to top-2 routing, while significantly reducing the load standard deviation (e.g., from 12.25 to 1.18 on MNLI). In addition, threshold-based dynamic expert activation reduces model parameters and provides a new perspective for mitigating load imbalance among experts.

## 1 Introduction

Transformer models are currently enjoying significant success in applications such as natural language processing (Radford et al., 2021), computer vision (Jiang et al., 2023; Zhu et al., 2024), and multimodal (Mustafa et al., 2022). And with the rapid development of Transformer models, the computational requirements have increased significantly. In general, scaling model size and training data is one of the direct and effective ways to reduce computational requirements, but it fails to better handle increasingly complex data, in this context, the Mixture of Experts (MoE) model provides a method that can expand model capacity and applicability without significantly increasing computational overhead, becoming one of the most effective methods to address the high computational demands of dense models (Mckinzie et al., 2024; Wu et al., 2024).

Mixture of experts is a sparse structure based on transformer (Lepikhin et al., 2020). It replaces the original FFN (Feed-Forward Network) layer with an expert layer, which consists of a gating network and various experts. Theoretically, experts can be combinations of different neural networks. Compared to traditional dense models, MoE achieves significant improvements in computational efficiency. MoE achieves sparsity mainly through the gating network, which routes each input to a specific subset of experts, reducing computational demands by activating only a small number of experts for training. However, MoE systems inherently face challenges of expert load imbalance caused by uneven training data distributions and divergent initial parameter preferences among experts. Expert load balance is a combination of the overall performance, efficiency, and robustness of the model. If some experts are over-activated while others remain idle for a long time, the actual effective number of parameters in the model will be lower than the theoretical value, leading to a waste of resources; frequently selected experts accelerated gradient updates, while underutilized counterparts stagnate in training, potentially trapping the optimization process in local minima and inducing a vicious cycle. Additionally, due to the limited capacity of experts, token overflow may occur under excessive input loads, resulting in partial data loss (Guo et al., 2024). Thus, expert load imbalance persists as a pervasive yet critical challenge in the MoE framework.

The current prevalent approach to mitigate expert load imbalance involves incorporating a load-balancing loss function into the optimization objective. This method quantifies activation frequency disparities among experts within each training batch using variance metrics, subsequently imposing penalty terms on the gating network’s outputs. During backpropagation, these penalties update model parameters to influence future token routing decisions. While theoretically sound, this methodology presents multiple implementation challenges:

1. Hyperparameter sensitivity: the auxiliary loss coefficient ( α ) requires manual calibration, where excessive values enforce artificial uniformity at the expense of model performance, while insufficient values compromise load balancing efficacy. Experiment indicates that optimal values exhibit phase-dependent variability—higher weights are preferable during the initial training phase, whereas later stages necessitate gradual reduction to prioritize task-specific optimization. Implementing dynamic α scheduling introduces nontrivial computational overhead.
2. Inherent data distribution bias: in scenarios with naturally imbalanced training data distributions, specific expert subsets demonstrate persistent activation dominance, and the balancing loss struggles to counteract such inherent biases.
3. Delayed feedback and scalability constraints: the impact of the load balancing loss function acts on the output weights of the gating network, requiring multiple iterations to update the parameters, then influences the next load distribution. Moreover, in the trillion-parameter scale model, the auxiliary loss function is used to make the expert utilization close to a uniform distribution.

Another issue is that in MoE models, inputs are typically allocated to a fixed number of experts. In tasks such as machine translation, MoE only improves BLEU (Bilingual Evaluation Understudy) by 0.5 compared to dense models with equivalent parameters, indicating that different inputs may require different numbers of experts for processing in specific downstream tasks (Xu et al., 2023).

To address the above issues, this paper proposes a dynamic routing load balancing model based on expert weights. The main contributions of our work are summarized as follows:

- We propose a dynamic routing load balancing algorithm based on expert weights, abandoning the load loss function and achieving load balancing through control expert weights directly. Specifically, during routing, input data generates the final routing score matrix through operations with expert weights, and the routing probability is obtained by normalizing the routing score. After each training batch, expert weights are continuously updated based on the activation in the previous batch, reducing the weight of highly loaded experts and increasing the weight of low-loaded experts, to balance the load among experts for the next routing. This method does not introduce a load-balancing hyperparameter, avoids the gradient effects of the loss function, and achieves load balancing among experts even in small-scale parameter models by directly adjusting expert weights without the need for multiple backpropagations, thereby improving the model’s adaptability.
- Our method can effectively alleviate the load imbalance issue in MoE models. In addition, dynamic routing achieves dynamic activation of expert subsets based on data complexity, improving computational efficiency and model performance. To verify the effectiveness of this method, experiments were conducted on standard datasets for natural language understanding. The experimental results show that our method outperforms traditional top-k methods in most tasks, achieving an average accuracy improvement of 0.7% compared to the top-2 routing model using loss functions on nine different datasets, and significantly improving the load imbalance among experts.

## 2 Related work

### 2.1 Mixture of experts model architecture

The Mixture of Experts model is a sparsely gated deep learning model consisting primarily of some expert models and a gating network (Xu et al., 2024). In this case, the MoE layer is composed of the gated network G(x) and multiple experts of the same network frame. Experts can be any identical or different models. For example, in a Transformer-based Mixture of Experts model, the expert network consists of several identical Feed-Forward Networks, and the MoE structure is typically placed after the self-attention sublayer to use the gating network to select the feed-forward networks within the Transformer block. This setup is because, as the model expands, the computational requirements for FFN increase (Zhang et al., 2024). In the 540B parameter PaLM model, 90% of the parameters are located in its FFN layer, so the MoE structure is placed after the attention layer to reduce the number of activated parameters (Chowdhery et al., 2023). Shazeer et al. (2017) introduced a sparse gating strategy that reduces computational overhead and achieves model sparsity by only computing the weighted sum of the outputs of the top few experts without significantly increasing the number of activated parameters in the model.

### 2.2 Top-*k* routing in mixture of experts models

The gating network is the core of the Mixture of Experts model and the method for achieving sparsity, responsible for matching tokens with experts. Top-*k* routing (Shazeer et al., 2017) is the most widely used routing algorithm; however, for tasks with different difficulties, it selects a fixed number of experts for activation. The number of activated experts, *k*, as a hyperparameter in the model, directly affects the model’s performance on different tasks and requires extensive ablation experiments to determine the optimal *k* value.

**Figure 1:**
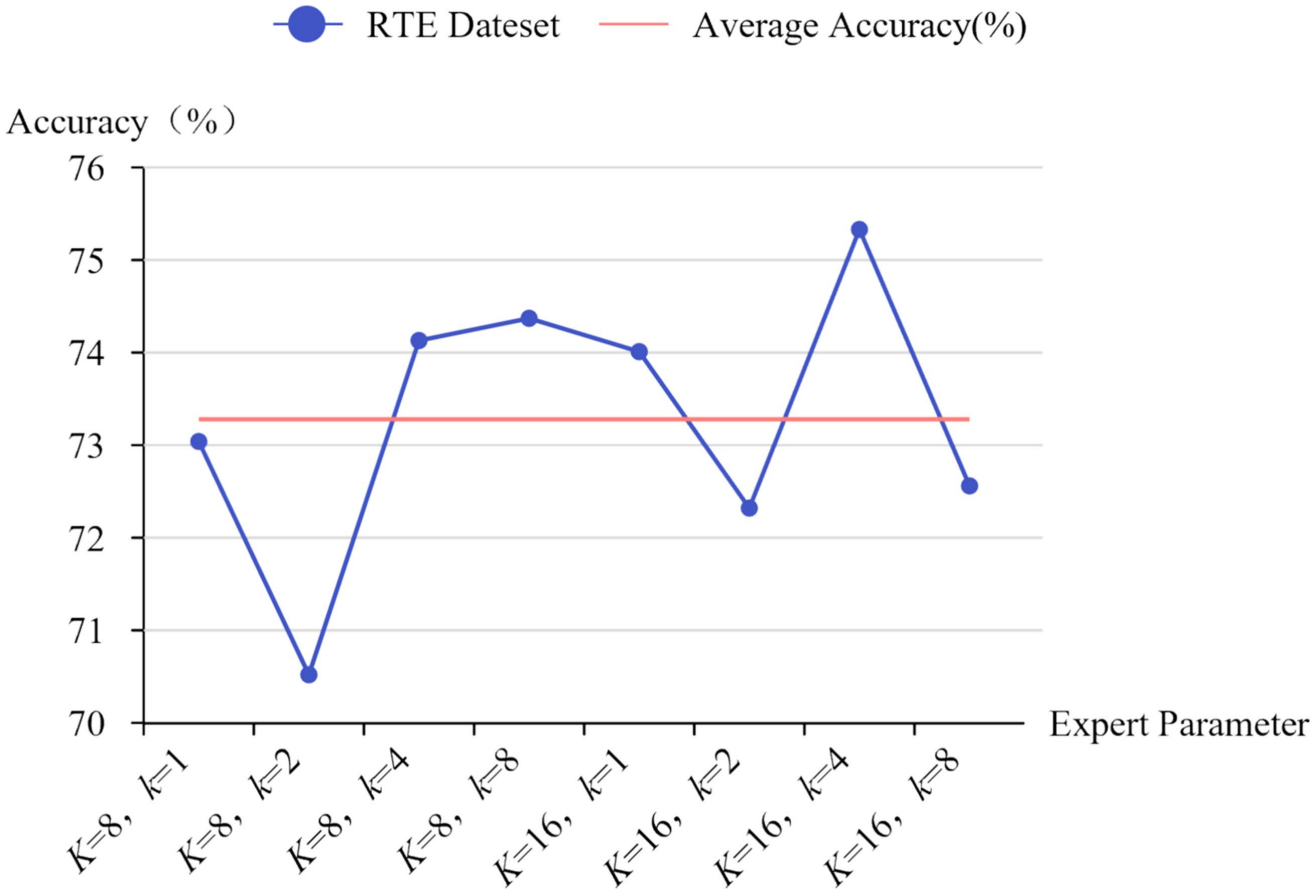
Impact of different expert settings on accuracy on the RTE dataset.

As shown in Figure 1 (where *K* is the total number of experts and *k* is the number of experts activated each time), different settings result in approximately 1–5% differences in model performance. As the model size increases, this limitation leads to wasted computational resources. Therefore, Huang et al. (2024) proposed a threshold-based dynamic routing method that can adjust the number of experts in the activated expert subset each time. After performing top-*k* routing, if the sum of the activation probabilities of the activated experts does not reach the activation threshold, it indicates that more experts are needed to jointly complete the task. This design dynamically allocates the number of experts based on the complexity of tokens. This method addresses the load balancing issue by using the loss function from Switch Transformer, adding the auxiliary loss for each MoE layer during training to the total loss of the model. Although the auxiliary loss can alleviate load imbalance to a certain extent, for dynamic routing, the number of activated experts is not fixed, and experts activated more frequently are more prone to load imbalance (Xie et al., 2024). Moreover, the contribution of each expert varies, and the loss function introduces additional hyperparameters and disturbs the gradient. Therefore, using a loss function requires extensive experiments to determine the optimal hyperparameter values to minimize its impact on the model.

When executing top-*k* routing, a batch input contains several tokens. For each token $x\in {\mathbb{R}}^{D}$ , D represents the dimension, *k* experts are selected by softmax function (Riquelme et al., 2021; Fedus et al., 2022a). The mathematical representation of the routing process is given by Equations 1–3.


(1) $$\mathrm{MoE}(x):\sum_{r=1}^{E}{\text{Gate}}_{r}(x)\cdot {\mathrm{MLP}}_{r}(x)\;\;$$

(2) $${\text{Gate}}_{r}(x)\cdots \mathrm{top}k(P)$$

(3) P=softmax(Wx+ε)

Where the gating weights linearly combine the outputs of *E* experts ${\left\{{\mathrm{MLP}}_{r}(x)\right\}}_{r=1}^{E}$ , where represents the routing probability between considering load balancing, ε is the load loss, and the expert weights $W\in {\mathbb{R}}^{E\times D}$ can be trained along with other network parameters. Equation 1 represents how each token selects the top *k* experts based on the softmax scores as well as the outputs of the MoE layer. Compared to the traditional multi-layer perceptron (MLP) layer, the MoE layer can reduce the computational cost by controlling the number of experts (Liu J. et al., 2024). Assuming each MoE layer processes T tokens $\{x_{1},\cdots ,x_{T}\}\subset {\mathbb{R}}^{D}$ , $X\in {\mathbb{R}}^{T\times D}$ X is the matrix composed of all tokens arranged by row. The MoE layer for processing a batch of data (containing T tokens) is defined as:


(4) $$\mathrm{MoE}(X):\sum_{r=1}^{E}\sum_{c=1}^{C}C_{X}[t,r,c]\cdot {\mathrm{MLP}}_{r}(X^{⊤}D_{X}[:,r,c])\in {\mathbb{R}}^{D}$$

In Equation 4, where ***D***_***X***_ and ***C***_***X***_ are the scheduling tensor (responsible for assigning different tokens to different experts) and the combination tensor (for linearly combining outputs across experts) of ***X***. *C* is the buffer capacity of each expert, which specifies the maximum number of tokens that each expert can process in a small batch, to efficiently utilize the hardware resources are generally C≪T , C=[T/E] or C=[2T/E] (Csaba et al., 2022). The output of the MoE layer is expressed as Equations 5, 6.


(5) $$C_{X}[t,r,c]:\{\begin{array}{l} {\text{Gate}}_{r}(x_{t})\;\;\text{if}\;t=c,\\ 0,\;\text{otherwise}, \end{array}\;\;D_{X}[t,r,c]:1(C_{X}[t,r,c]>0)$$

(6) $$\begin{array}{l} \mathrm{MoE}(X)=\sum_{r=1}^{E}\sum_{c=1}^{C}C_{X}[t,r,c]\cdot {\mathrm{MLP}}_{r}(X^{⊤}D_{X}[:,r,c])\\ \;=\sum_{r=1}^{E}C_{X}[t,r,t]\cdot {\mathrm{MLP}}_{r}(X^{⊤}D_{X}[:,r,t])\\ \;=\sum_{r=1}^{E}{\text{Gate}}_{r}(x_{t})\cdot {\mathrm{MLP}}_{r}(x_{t}) \end{array}$$

The core of the MoE layer is to map the input ***X*** to two tensors and obtain the final expert layer output through calculations. The whole routing process can be simplified as $\text{Router}:X\to (D_{X},C_{X})$ (Liu T. et al., 2024). In the routing process, ***W***, as the expert weight, can directly affect the allocation of the token. Before executing the top*-k* router, the similarity matrix between the expert and the token is first constructed Πsoftmax , as shown in Equation 7:


(7) $$\Pi \;\text{softmax}:\text{softmax}(\mathrm{XW}+L_{\text{Balance}})$$

In the absence of any regularization constraints, the maximum value of each row of the final routing score matrix may be concentrated at certain indexes, i.e., most of the tokens are routed to a few fixed experts, which makes some experts underutilized. Simultaneously, due to the limited expert capacity, subsequent tokens may be lost. To prevent this from happening, the current work is through the addition of auxiliary loss ${ℒ}_{\text{Balance}}$ to mitigate (Lepikhin et al., 2020; Fedus et al., 2022b), for a sequence of length T, the auxiliary loss is defined as the Equation 8:


(8) $$\begin{array}{l} {ℒ}_{\text{Balance}}=\alpha \sum_{i=1}^{E}f_{i}P_{i},\\ \;\;\;f_{i}=\frac{E}{\mathrm{kT}}\sum_{t=1}^{T}1(\text{Token}\;t\;\text{selects expert}\;i),\\ \;\;P_{i}=\frac{1}{T}\sum_{t=1}^{T}s_{i,t}. \end{array}$$

where E is the total number of experts, *k* is the number of experts selected by each token, $s_{i,t}$ is the routing score of the *t*th token at expert i, $f_{i}$ represents the routing score of tokens routed to expert i, $P_{i}$ represents the average routing score of expert i, and is the hyperparameter controlling the strength of the auxiliary loss. Although adding an auxiliary loss function can alleviate the load imbalance, the size of the hyperparameter α affects the overall performance of the model; a smaller α will lead to routing collapse, which affects the model efficiency and may result in some experts not being able to learn or utilize adequately; while a larger α will keep the load balance in a controlled state and significantly reduce the model performance (You et al., 2021). Therefore, our method directly controls expert weights to avoid introducing loss functions to solve the load-balancing issue among experts (Li et al., 2025).

### 2.3 Dynamic routing

Traditional top*-k* routing limits the number of activated experts, leading to unnecessary waste in some cases (Yang et al., 2024). Experiments have shown (Clark et al., 2022; Fan et al., 2024) that the performance of MoE models can be significantly different depending on the value of k in the top*-k*, and thus a large amount of computational resources are needed to verify the optimal value of k for different downstream tasks; secondly, top*-k* gating methods assume that each token must activate the same number of experts, which does not satisfy the task needs in practice. A fixed number of experts can produce lower confidence when handling certain difficult tasks, affecting output results.

Guo et al. (2024) proposed DYNMOE, an algorithm that can automatically determine the number of activated experts during both training and testing. By modeling the gating mechanism as a multi-label classification problem, treating each expert as a separate category, and independently computing the gating score for each expert, all experts with scores exceeding the threshold are activated. This allows different tokens to activate different numbers of experts. When a token chooses not to activate any existing experts, it adds a new expert and deletes any unused experts. However, without constraining the maximum number of activated experts, tokens may activate all experts or only a few specific experts. Activating all experts can lead to high similarity and insufficient specialization among experts, while activating specific experts can cause severe load imbalance. Although DYNMOE also adds an auxiliary loss function to alleviate these issues, computing losses for each token routing, adding and deleting experts, introduces additional computational costs and greater memory requirements, posing challenges for model training and testing.

## 3 Method design

Addressing the limitations of top-*k* routing, Huang et al. (2024) argued that traditional routing ignores the variation in difficulty among different inputs and activates a fixed number of experts at each layer of the Transformer, ignoring differences in cross-layer representations. Therefore, different numbers of experts are needed for different layers. Under these conditions, they designed a threshold-based dynamic routing strategy. By determining whether the currently activated number of experts reaches the threshold, more experts are activated to increase the reliability of token processing. This method first sorts the routing probability values P to get the sorted index **I,** finds the smallest set of experts S that cumulatively exceeds the threshold *p*, where *p* is a hyperparameter with a value range of [0, 1] and is set to 0.4 in the original experiment. A larger *p*-value indicates that more experts need to be activated. Load balancing among experts adopts the method proposed by Fedus et al. (2022b), where the hyperparameter *α* requires experimental verification and cannot adapt to different downstream tasks.

**Figure 2:**
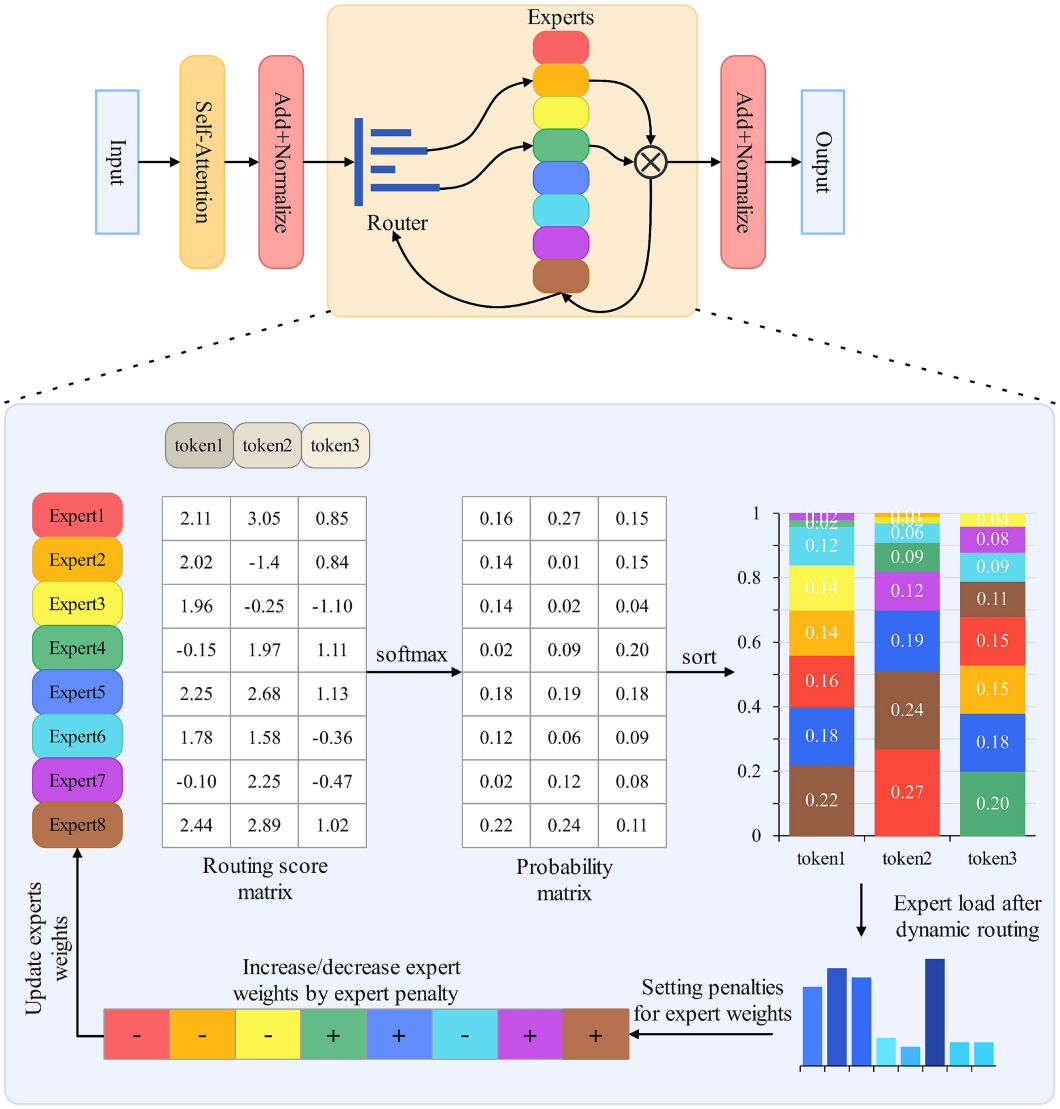
Different routing results by adjusting expert weights.

In this paper, we adopt the dynamic routing strategy proposed by Huang et al. and design an expert weight-based dynamic routing method, primarily optimizing load balancing. Traditional load balancing is achieved by adding auxiliary loss functions. Since loss functions introduce additional hyperparameters that need to be tuned to fit the current task, and improper selection of hyperparameters may cause the model to pay too much attention to load balancing (Nguyen et al., 2023), which will weaken the performance of the main task, so this paper, from the principle of routing algorithms in the MoE gated network, alleviates the issue of load imbalance through the adjustment of the expert weights. Specifically, after routing each batch of data, a penalty term is set based on the load situation of the current round of training, reducing the weights of highly loaded experts and increasing the weights of low-loaded experts. The advantage of this method is that it avoids introducing additional auxiliary loss functions, eliminating the impact of hyperparameters on the model’s handling of different downstream tasks. Models activate experts by routing probabilities, where expert weights play a key role in the routing process, and different weights determine the subset of experts activated during dynamic routing (Fedus et al., 2022a, 2022b). The routing principle is shown in Figure 2, which illustrates how the Mixture of Experts model selects experts through the generated similarity matrix for top*-k* routing.

Threshold-based dynamic routing can be regarded as a variant of top*-k* routing, both selecting the most suitable experts by computing the routing score matrix through operations between expert weights and tokens. The expert weight is a multi-dimensional vector, with each dimension representing the degree of specialization in different fields. A higher weight represents a greater proportion of the expert’s processing results. The dynamic routing design idea is to activate a corresponding number of experts based on task difficulty. Dynamic expert activation improves model efficiency but also brings new issues: for example, in the bottom layer of the model and the early stage of training, the dynamic routing will activate more experts to ensure the reliability of the output, more experts means higher activation rate is also more prone to imbalance load (Folino et al., 2024). To better achieve load balancing, this paper directly adjusts expert weights based on the load situation. The advantage of this design is that expert weights play a decisive role in routing. Each token needs to compute with expert weights to construct the routing matrix. After routing each batch of data, the load situation of all experts is counted, and the weights are updated through an algorithm (Jiang et al., 2024), the reason for not needing to pay attention to the load of the experts frequently is that in the language task, the data in each batch basically come from the same sentence or paragraph, and there is a connection between the front and back, if it is too frequent, the expert load will be adjusted according to the load. If expert load balancing is pursued too frequently, it will easily lead to the loss of contextual connection, violating the natural division of labor mechanism of expert models, and additionally consuming computational resources.

For the input set ***X***, dynamic routing is performed first, and a larger threshold is set for dynamic routing. At the initial stage of model training, randomly assigned tokens are given to the experts, and the expert weights will gradually show specialization as the model is trained together, so a larger threshold is needed to allow more experts to participate in the training. In addition, this also helps the model acquire more shallow representations for subsequent deep expert activation. After obtaining the routing probability of each token, it is sorted in descending order. The expert subset whose cumulative probability exceeds the threshold is taken as the routing experts for this round. Subsequently, the number of tokens assigned to each expert is counted, and the average number is determined. The load penalty is calculated, and the load weight of each expert at the token level is computed. The expert weights are then updated using Equation 9. Algorithm 1 describes the flow of this method. By optimizing the load balancing scheme in dynamic routing, this paper achieves dynamic expert selection based on input while balancing the task load among experts, leveraging model advantages to improve efficiency.

**ALGORITHM 1:**
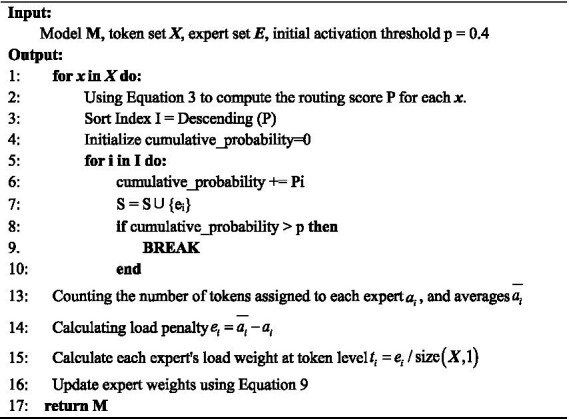
Load balancing algorithm based on expert weights.

Since ε represents the amount of load loss in Equation 3, the load balancing scheme adopted by Fedus et al. makes $\varepsilon ={ℒ}_{\text{Balance}}$ , our scheme proposed in this paper does not use a loss function, so makes ε=0 in Equation 3, the set of all activated experts is obtained at the end of the current batch of data routing, $e_{i}=\bar{a_{i}}-a_{i}$ represents the number of tokens processed by the i-th expert that is more (less) than that in the balanced state ( $a_{i}$ is the number of assigned tokens for each expert and $\bar{a_{i}}$ is the ( $t_{i}$ is the number of assigned tokens per expert, is the average number of assigned tokens per expert), and represents the load weight deviation of the *i-*th expert at the token level, accordingly, by mapping the deviation at the token level to the expert weight level, and updating the existing expert weights through Equation 9 to achieve the load balancing for the next route.


(9) $$w_{i}=w_{i}+(w_{i}/\sum_{i=1}^{E}w_{i})\times t_{i}$$

Our goal is to use dynamic routing to influence the expert weights in the next round through load weight deviations at the token level, so that experts can include load constraints from the previous round in the next round of routing calculations. In the expert activation probabilities of the next round $P=\text{softmax}(w_{i}x)$ , after being updated by Equation 9, the expert weights include the load conditions from the previous round. The weight update directly affects the activation probability of the expert for the input token. Among them, $w_{i}/\sum_{i=1}^{E}w_{i}$ represents the proportion of the current expert weight in the total weight. When $t_{i}$ is higher than the load average, it takes a negative value, reducing the current expert weight and suppressing activation in the next round. Conversely, it promotes load balancing between experts.

In the algorithm design, expert weights are updated after each batch routing because, in language tasks, tokens are somewhat correlated. Responding to load balancing in real-time would consume significant computational resources and affect the task accuracy of language models. For example, pronouns in the sentence often require more experts to process them collaboratively, and at the same time, the participating experts should also include those who deal with the referenced pronouns. This load imbalance is due to the fact that special tasks activate more experts, thus increasing the confidence of the model to handle some of the difficult tasks.

## 4 Experimental design

Based on the aforementioned design, the Mixtral-8×7B is adopted as the core architecture of the model, which is a Transformer-based mixture of experts model consisting of 8 feed-forward networks (i.e., experts) per layer, with the parameter size of 7B for each expert, and the number of hidden layers and attention headers both being 32. Experiments are conducted on a natural language understanding dataset; the main focus is on the load balancing of the model, the average number of activations of the experts, and the accuracy of each sub-task were evaluated. In multiple benchmark tests, its performance reached or surpassed that of Llama 2-70B (Touvron et al., 2023), especially demonstrating outstanding capabilities in mathematics and multilingual understanding tasks.

### 4.1 Dataset

Natural Language Understanding (NLU) is a subfield of Natural Language Processing (NLP) that focuses on making computers understand the meaning of human language (Clark et al., 2022). The validity of the model was verified on a publicly available dataset. In this paper, we use the multi-tasked Natural Language Understanding dataset GLUE (Wang et al., 2018) (General Language Understanding Evaluation) created from New York University, University of Washington, and other institutions. GLUE contains nine Natural Language Understanding tasks, all in the language of English. It is used to evaluate the performance of the model in various existing NLU tasks. The nine tasks of GLUE involve multiple tasks, such as natural language inference, textual entailment, sentiment analysis, semantic similarity, etc. GLUE has nine tasks, namely CoLA, SST-2, MRPC, STS-B, QQP, MNLI, QNLI, RTE, and WNLI, which can be categorized into three types, namely, Single-sentence classification tasks, similarity tasks, and inference tasks.

Single-sentence classification tasks include: sentiment classification (SST-2), judging whether it is grammatical or not (CoLA), all of which can be abstracted into a binary task to judge whether the sentence is (or is not) grammatical; similarity tasks include: judging whether two sentences express the same meaning (MRPC, QQP), and judging the relevance of the two sentences to each other (STS-B); inference tasks include: whether the sentences are semantics are contradictory and implicit (MNLI, RTE), whether there is an answer to a certain question in a sentence (QNLI), and which object is referred to by a pronoun (WNLI).

### 4.2 Model structure and parameter settings

Mixtral-8 × 7B is based on the Transformer architecture (Jiang et al., 2024) with up to 32 k token length of processing context and feedforward blocks are replaced by MoE layers. The number of Transformer layers is set to 32, the embedding size for the feedforward network (FFN) is 4,096, the number of Q (query) vectors is 32, and the number of K (key) and V (value) vectors is 8. Each layer consists of 8 experts, with each expert having 7B parameters. The top two experts are activated each time, and the SwiGLU activation function is used in the FFN layers. The threshold *p* is a hyperparameter with an initial value of 0.4. All other learnable parameters are randomly initialized before pretraining with a standard deviation of 0.006. In the GLUE dataset, for tasks with relatively large amounts of training data (MNLI, SST-2, QQP, QNLI), the number of epochs is set to 6. For tasks with limited data (CoLA, STS-B, MRPC, RTE, WNLI), the number of epochs is increased to 10. The warmup steps value is set to 16. Additionally, due to the limited training data of only around six hundred samples in WNLI, the batch size for this task is set to 16 separately.

### 4.3 Baseline

In the experimental design, we set up one dense model and two Mixture-of-Experts (MoE) models using top-1 and top-2 routing, respectively. The dense model follows the standard Transformer architecture, where each Transformer layer consists of a multi-head attention layer and a standard feedforward network (Vaswani et al., 2017). The baseline MoE model achieves load balancing by incorporating auxiliary loss functions. Specifically, the top-1 routing adopts the Switch Transformer model (Fedus et al., 2022a, 2022b), while the top-2 routing employs the Gshard model (Chen et al., 2023). For a fair comparison, all three baseline methods are configured identically to the method proposed in this paper, and are trained with learning rates set to {1e-3, 2e-3, 5e-3}, although only the best experimental results are used for comparison. For the baseline MoE models, the loss function proposed by Fedus et al. is used, which involves the hyperparameters α=0.001 .

### 4.4 Evaluation indicators

For the three types of downstream tasks in the GLUE dataset, the accuracy rate (ACC) was used as an evaluation metric. The calculation method for accuracy rate is shown in Equation 10.


(10) $$\mathrm{ACC}=\frac{\mathrm{TP}+\mathrm{TN}}{\mathrm{TP}+\mathrm{TN}+\mathrm{FP}+\mathrm{FN}}$$

Where: TP (True Positives) refers to true cases, FP (False Positives) refers to false positive cases, FN (False Negatives) refers to false negative cases, and TN (True Negatives) refers to true negative cases. Load balancing is evaluated using the standard deviation σ of the proportion of tokens $P_{i}$ received by each expert $E_{i}$ as an evaluation index of load balancing, with the smaller standard deviation representing the more balanced load. Evaluation metrics for load balancing are shown in Equation 11.


(11) $$\sigma =\sqrt{\frac{1}{E}\sum_{i=1}^{E}{\left(P_{i}-\bar{P}\right)}^{2}}$$

## 5 Results

The experiment evaluates the effectiveness of the models based on the training and test data provided by the GLUE dataset, using the test accuracy of each task as the metric. Table 1 presents the test accuracies of four models, each with 8 experts, on three different types of downstream tasks in the GLUE dataset, employing various routing strategies and auxiliary functions.

**Table 1:** Accuracy of the four models on the GULE dataset.

Models	Single-sentence classification tasks			Similarity tasks			Inference tasks			Average precision
	COLA	SST-2	MRPC	STS-B	QQP	MNLI	QNLI	RTE	WNLI	
dense	57.1	92.3	87.5	89.1	90	80.7	88.9	**75.6**	65.6	80.75
top-1	64.2	93.3	86.3	88.2	91.3	81.1	92.3	73.9	65.4	81.77
top-2	64.4	94	**87.7**	88.2	92.1	83.8	92.9	75.1	65.7	82.65
**Ours**	**65.1**	**94.7**	86.7	**89.3**	**93.2**	**86.5**	**93.2**	74.4	**66.9**	**83.33**

Bold indicates the best result.

The experimental results show that the MoE model outperforms traditional dense models in the vast majority of tasks. This is because the MoE model can increase model capacity under the same parameter settings, and the number of parameters activated at each step is significantly smaller than that of dense models, thereby reducing inference costs. Compared with other models, the method proposed in this paper achieves an average accuracy rate 6.1% higher than that of dense models and 0.7% higher than the currently widely used top-2 routing, indicating that the proposed model is applicable to general natural language understanding tasks and can achieve good performance. However, on the RTE and MRPC datasets, the accuracy is slightly lower than that of top-2 routing, indicating that the proposed model cannot fully leverage the advantages of dynamic routing in scenarios with limited data. For datasets with sufficient data (such as QQP and MNLI), the model can improve accuracy by over 1%. Both MRPC and QQP suffer from imbalanced positive and negative sample distributions. In QQP, negative samples account for 63% and positive samples account for 37%. By using the load balancing method proposed in this paper, the model can avoid token loss caused by selecting only a portion of experts in scenarios with imbalanced positive and negative sample distributions. Therefore, it can achieve better results than top-2 in classification tasks, demonstrating the robustness of this method to imbalanced sample distributions in large datasets.

The model performs poorly on small datasets, primarily because the training datasets for RTE and MRPC contain only 2,500 and 3,700 examples, respectively, with negative examples accounting for as much as 65% of the total. To further investigate the impact of small datasets on model performance, we conducted the following comparative experiments. First, we gradually reduced the size of the training set, using 100, 75, and 50% of the training set data to train the model. Under the setting of batch_size = 16, we calculated the average variance of the expert load for the first layer, the eighth layer, and the sixteenth layer, with ACC as the evaluation metric. The experimental results are shown in Table 2.

**Table 2:** Load variance and accuracy of the expert layer under different dataset sizes

	1st-layer	8th-layer	16th-layer	ACC
RET (100%)	1.66	1.51	1.74	74.47
MRPC (100%)	1.17	1.28	1.20	86.71
RET (75%)	1.71	1.98	2.33	72.92
MRPC (75%)	1.65	2.02	2.46	85.34
RET (50%)	1.90	3.19	3.67	69.86
MRPC (50%)	1.93	2.79	4.17	82.07

As the training data decreased, the model accuracy also deteriorated gradually, with the highest decrease of 3.27%. From a routing perspective, a core feature of MoE is that different experts focus on different input subspaces. However, in small-sample datasets, the router randomly distributes the limited samples to multiple experts in the early stages, resulting in smaller effective sample sizes for each expert. Due to insufficient sample size, the experts cannot train stable patterns, leading to inaccurate routing of tokens to relevant experts in subsequent stages. We then adjusted the batch size during training, setting it to 8, 16, and 32, and included a large dataset with sufficient data as a comparison (QNLI). The experimental results are shown in Table 3. Under the condition of a constant epoch, the small dataset performs best when batch_size = 16. This is because a smaller batch size increases the probability of all samples within a batch belonging to the same category, leading to an increase in the activation frequency of certain experts. Dynamic routing then reduces the weight of these experts, affecting the results of subsequent routing rounds. A larger batch_size setting reduces the number of model iterations, resulting in poor generalization performance. Through the above comparative experiments, it was verified that the primary reason for the poor performance of the model on small datasets is the limited data volume and uneven data distribution. However, by adjusting the batch size, the issue of poor model accuracy can be mitigated, thereby enhancing the model’s adaptability to limited data.

**Table 3:** The impact of different batch sizes on model accuracy.

Dataset	ACC		
	Batch_size = 8	Batch_size = 16	Batch_size = 32
RET (100%)	74.13	**74.91**	74.44
MRPC (100%)	86.76	**87.11**	86.73
QNLI (100%)	92.50	93.04	**93.21**

**Figure 3:**
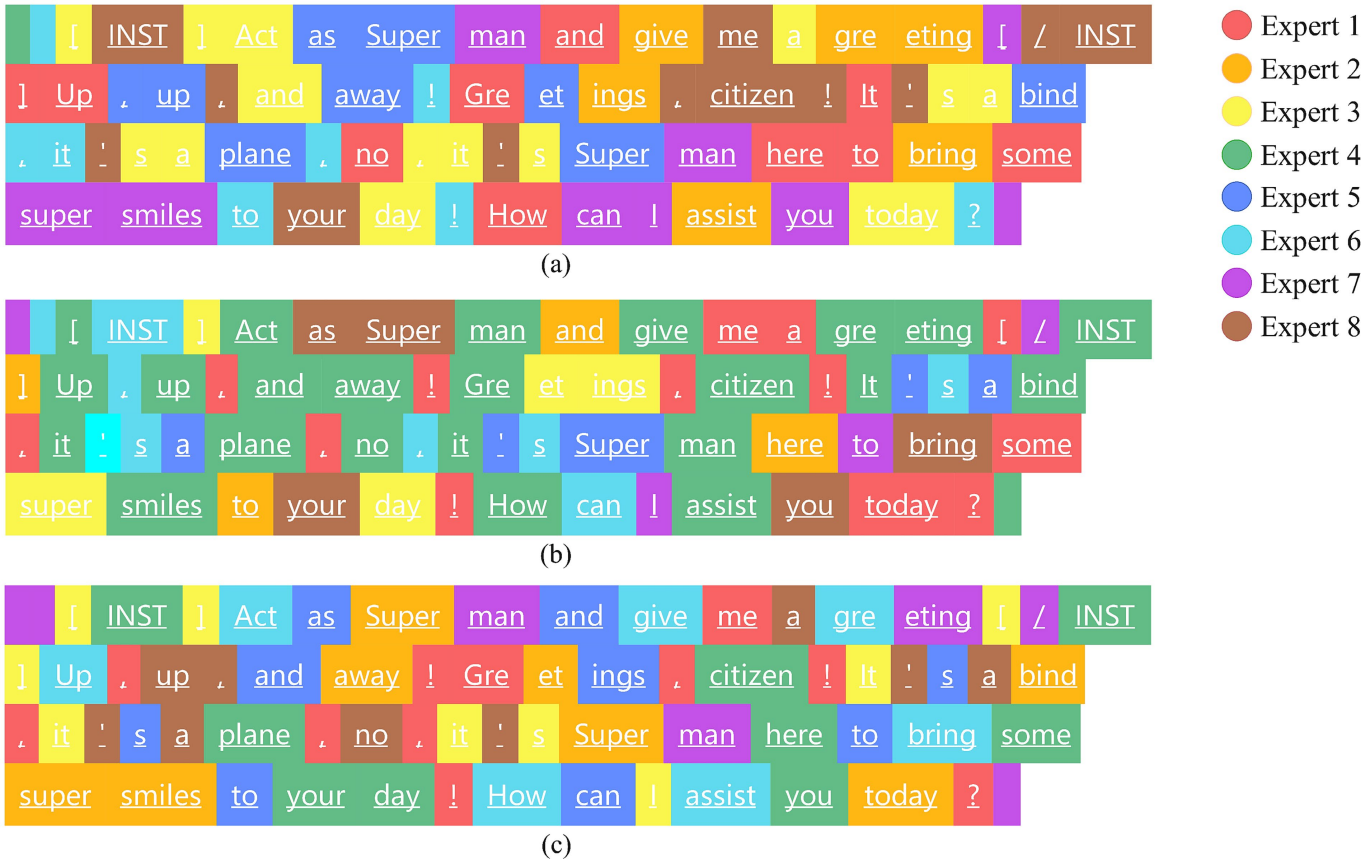
Routing visualization results for different layers of MoE for the same sentence. **(a)** and **(b)** show the routing results in the first and last MoE layers respectively, while **(c)** shows the routing result in the last layer with dynamic routing.

Jiang et al. found that for higher levels in the MoE model, the continuous allocation phenomenon is significantly higher than the random allocation (Jiang et al., 2024). This implies that without load balancing control, the model will be more likely to activate only a few experts, which seriously affects the training and reasoning of the model, and results in a waste of resources of free experts. The expert allocation of the first MoE layer and the last MoE layer in the Mixtral-8×7B model for the same sentence is verified by visualizing the expert allocation of the first MoE layer and the last MoE layer in the Mixtral-8 × 7B model as shown in Figure 3, which verifies that the method proposed in this paper is effective in balancing the load of high-level expert effectiveness.

Figures 3a,b shows the allocation of eight experts to the same sentence in the first and last layers of MoE, respectively, from which it is found that the last layer is more prone to successive assignment of experts in token assignment, and the activation frequency of each expert is counted, and there is a serious load imbalance compared to the first layer, in which the activation rate of the expert 4 grows from 1.45 to 34.78%, and that of the experts 2 and expert 4 is only 5.8%. Figure 3c represents the load of the last layer of experts after adopting the load-balancing scheme of this paper. The standard deviation value of the expert load is 1.76, compared to 9.33 in Figure 3b. Furthermore, the variance of expert load was analyzed for other layers (from Layer 1 to Layer 7), and it was found that the proposed method outperformed the loss function method proposed by Fedus et al. for all layers except the first two. This is because both methods use random allocation in the first layer, leading to similar performance and achieving uniform allocation. Therefore, the method designed in this paper has little impact on adjusting expert weights in the first layer. However, as the number of MoE layers increases, the loss function fails to effectively handle the consecutive allocation phenomenon, resulting in worsened load imbalance with increasing layers.

**Figure 4:**
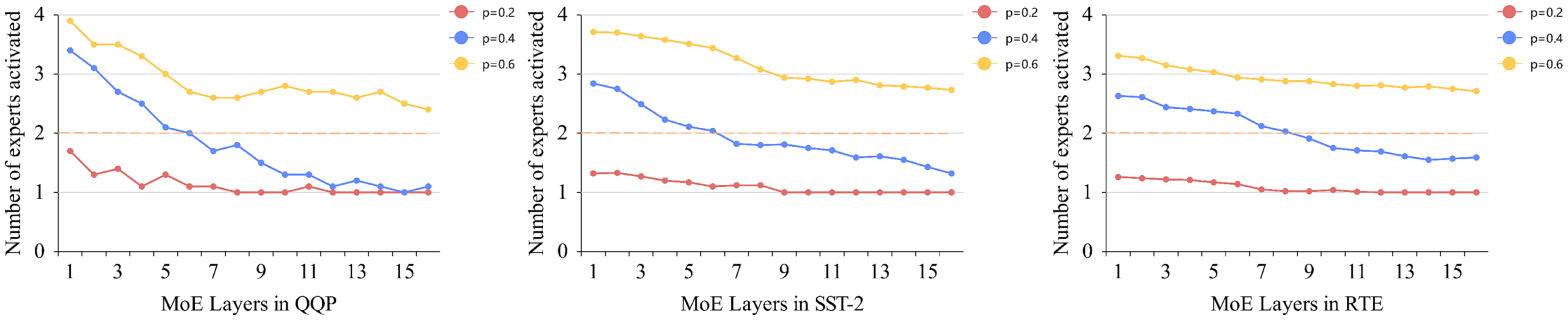
Average number of activated experts per MoE layer at different thresholds.

To verify the impact of thresholds on the model in dynamic routing, we selected the QQP, SST-2, and RTE datasets from GLUE based on their data volume sizes. RTE based on the size of the data. The sizes of their training sets are approximately 360 k, 67 k, and 3 k, respectively, and the threshold sizes are set to 0.2, 0.4, and 0.6, respectively. By statistically analyzing the number of expert activations and experimental results under different thresholds, we verify the reasonableness of the thresholds. Figure 4 shows the number of expert activations in the MoE layer using dynamic routing across the three datasets at different thresholds, with dashed lines indicating the number of top-2 routed activations. As the dataset size decreases, the number of expert activations at different thresholds within the same dataset also decreases, and the rate of decrease becomes smaller. This is because, as the dataset size decreases, it becomes increasingly difficult to quickly form highly specialized experts. The dynamic routing strategy must balance load balancing while meeting activation threshold requirements. Therefore, in larger datasets, the proposed dynamic routing strategy is highly effective in reducing the number of expert activations. During the early stages of model training or in shallow layers, experts have not yet reached a specialized level, and the distribution of routing probabilities is often relatively uniform. When the threshold is set to *p* = 0.2, the threshold is easily met, and even if all experts have the same activation probability, only two experts are needed to reach the threshold. Therefore, as training progresses or in deeper layers of the model, a low threshold causes routing to degrade into traditional top-1 routing, and this phenomenon becomes more pronounced as the dataset size decreases. As the threshold increases, dynamic routing activates the corresponding number of experts based on routing probabilities to promote specialization. In the three datasets mentioned above, the number of activated experts in deeper layers is significantly lower than in shallower layers. This is because, after training, experts with higher specialization levels can meet the threshold constraint without activating additional experts for computation. Table 4 shows the experimental results of dynamic routing on datasets of different sizes at different thresholds. In all three datasets, the setting of *p* = 0.4 yields the best results. Thus, in this task also validates the effectiveness of the *p*-value set to 0.4 by Huang et al.

**Table 4:** The impact of different threshold settings on experimental results.

Dataset	ACC		
	*p* = 0.2	*p* = 0.4	*p* = 0.6
QQP	91.7	**93.2**	92.9
SST-2	89.2	**94.7**	93.5
RET	73.0	**74.4**	74.3

Bold indicates the best result.

In general, the performance of a model for downstream tasks usually depends on the number of parameters (Gao et al., 2024). For example, top-2 routing showed significantly better performance than top-1 routing in experiments. Zhou et al. (2020) demonstrated that deeper representations of the model are prone to overfitting phenomena, and therefore, good shallow representations are more valuable than complex deep representations (Sajjad et al., 2023). These two phenomena indicate that both the number of parameters and shallow representations can effectively improve model performance. The scheme proposed in this paper achieves comprehensive feature extraction in the shallow layers through multiple experts, and by influencing expert weights and dynamic routing, activates fewer experts in the deep layers, thus achieving a balanced load among experts while reducing the number of model parameters.

WNLI is a dataset for a binary classification task, which includes 634 training samples and 146 test samples. The training set has a balanced distribution of positive and negative samples, while 65% of the test samples are negative. The main reason for the consistent number of dynamically activated experts is that for more challenging reasoning tasks, the model will activate more experts to participate. Limited training data, uneven distribution of positive and negative samples, and task difficulty all contribute to the model not having an advantage in the average number of experts activated for this task. In the top layers of the model, around two experts are also activated, and due to insufficient data distribution and quantity, the scheme degenerates into top-2 routing in terms of average activation number. Through experiments on nine datasets, the proposed scheme in this paper not only activates fewer experts (i.e., fewer parameters) while achieving load balancing, but also obtains better shallow representations by activating more experts in the lower layers of the model. As the number of layers increases, the number of experts activated per token gradually decreases, effectively preventing the model from becoming overly complex and reducing unnecessary computations. Except for a few datasets, the proposed scheme achieves better results than top-2 routing.

## 6 Ablation experiment

**Figure 5:**
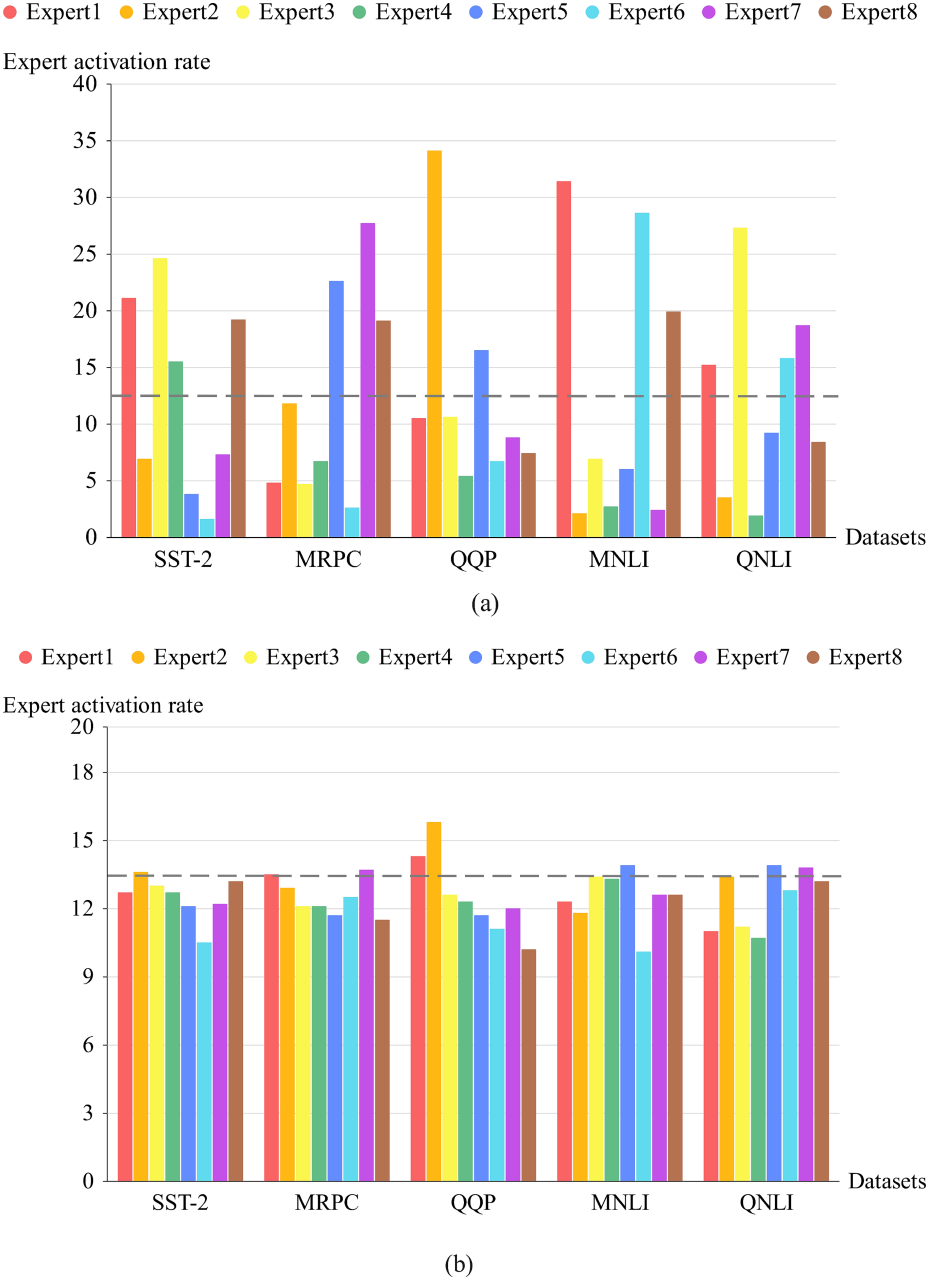
Expert activation rate for two load losses under dynamic routing. **(a)** shows the results of using a loss function, **(b)** shows the results of using dynamic routing.

The ablation experiment selects five datasets from GLUE according to the size of the data volume to verify the effect of the method proposed in this paper on the load balancing of experts. The load conditions of eight experts during the training process were recorded, demonstrating that the proposed load balancing scheme outperforms the method using a loss function on the same datasets. The experimental results are shown in Figure 5. Figure 5a shows the activation rates of the eight experts on different datasets when the loss function is used to achieve load balancing, and it can be seen that more serious load balancing occurs in each dataset. The gray dashed line represents the ideal expert activation rate. Load imbalance is more severe in some datasets with larger data volumes (e.g., QQP and MNLI). This is because load balancing loss is achieved by regularizing the selection probability distribution of the gating network, typically using softmax or other normalization functions, whose gradients can become very small or very large near extreme values (Wang et al., 2024). This gradient issue may cause the selection probability of certain experts to increase rapidly, exacerbating the imbalance. Secondly, after adopting dynamic routing, the number of experts activated each time is not fixed. At the beginning of training, the model activates more experts to obtain shallow representations, as shown in Figure 4. This reduces the weight of the penalty term intended to improve load imbalance among experts. Therefore, the loss function cannot effectively improve expert load balancing, resulting in the activation rate of some experts being much higher than the ideal value throughout the training process. Figure 5b shows the dynamic routing strategy using the proposed expert weight-based dynamic routing scheme, which achieves good load-balancing effects on different datasets. By directly modifying expert weights, it avoids the gradient influence brought by the loss function and does not increase the load loss as the number of training batches increases. It also performs well on larger datasets, verifying that the proposed method can effectively alleviate load imbalance among experts.

For the above five datasets, the standard deviation of expert activation under two kinds of load loss is calculated as a criterion to verify the effectiveness of the model, as shown in Table 5. The standard deviation is reduced on all five datasets, with the most significant improvement in load imbalance observed on the MNLI dataset. Accordingly, in Table 1, it can be seen, the experimental accuracy of the load balancing algorithm proposed in this paper on the MNLI dataset has been improved by 2.7%, which is the largest accuracy improvement in all the tasks. This also confirms that the load imbalance affects the accuracy of the model and the training effect. In addition, our method employs dynamic routing; the experimental results demonstrate that this approach enables the model to activate fewer experts (parameters), reducing the computational requirements of the model while maintaining load balancing among experts. This further optimizes the Mixture of Experts (MoE) model.

**Table 5:** Standard deviation of expert activation for two load losses.

Dataset	Standard deviation of expert activation	
	Based on the loss function	Based on expert weights
SST-2	8.68	0.95
MRPC	9.48	0.81
QQP	9.36	1.76
MNLI	12.25	1.18
QNLI	8.42	1.32

In MoE models, sparsity is achieved through the gating network G(x) without significantly increasing computational costs. Although load imbalance directly affects the optimal performance of the model when dealing with large datasets and complex tasks, in some cases, load imbalance may be meaningful. For example, if a certain batch of input data is mostly of one type, the hybrid expert model was originally designed with the intention that different experts would process different kinds of inputs Instead of evenly distributing the same kind of data to different experts (Xie et al., 2024), the forced equalization may violate the natural division of labor mechanism of the model. The current load balancing mechanism does not take into account the imbalance of the training data itself, and only achieves the average load balancing state through the expert weights setting, and requires the uniform distribution of all kinds of training data in the model training to avoid the load brought by the distribution of the data itself. In the model training, all types of training data are required to be evenly distributed to avoid the load imbalance caused by the distribution of the data itself. If the data are naturally imbalanced distributed, the pursuit of absolute equalization may affect the model effect (Yan et al., 2024). Future work will focus on more realistic scenarios, addressing how to avoid the aforementioned issues when data itself is imbalanced. Additionally, experiments have shown that the model’s performance is not ideal when testing on datasets with uneven label distributions (Hao et al., 2025). Further research is needed to address load balancing issues arising from small sample sizes, uneven sample distributions, and other related challenges.

## 7 Summarize

This paper proposes a dynamic routing algorithm based on expert weights for load balancing, which adjusts expert weights based on the load situation among experts during the previous routing to influence the next routing allocation. The advantage of this method lies in avoiding the training settings of hyperparameters in the loss function and the impact of gradient changes on expert loads. Experimental validation demonstrates that the dynamic routing algorithm based on expert weights not only improves task accuracy but also reduces the model’s parameter count compared to top-*k* routing, while achieving good load-balancing effects. Especially in datasets with sufficient data, this routing algorithm can better leverage its advantages.

## Data Availability

The raw data supporting the conclusions of this article will be made available by the authors, without undue reservation.
